# Does the “obesity paradox” exist after transcatheter aortic valve implantation?

**DOI:** 10.1186/s13019-022-01910-x

**Published:** 2022-06-13

**Authors:** Zeng-Rong Luo, Liang-wan Chen, Han-Fan Qiu

**Affiliations:** Key Laboratory of Cardio-Thoracic Surgery, Department of Cardiovascular Surgery and Cardiac Disease Center, Union Hospital, Fujian Medical University, Fujian Province University, Fuzhou, 350001 People’s Republic of China

**Keywords:** Aortic stenosis, TAVI, Body mass index, Intubation time

## Abstract

**Background:**

Transcatheter aortic valve implantation (TAVI) for symptomatic aortic stenosis is considered a minimally invasive procedure. Body mass index (BMI) has been rarely evaluated for pulmonary complications after TAVI. This study aimed to assess the influence of BMI on pulmonary complications and other related outcomes after TAVI.

**Methods:**

The clinical data of 109 patients who underwent TAVI in our hospital from May 2018 to April 2021 were retrospectively analyzed. Patients were divided into three groups according to BMI: low weight (BMI < 21.9 kg/m^2^, n = 27), middle weight (BMI 21.9–27.0 kg/m^2^, n = 55), and high weight (BMI > 27.0 kg/m^2^, n = 27); and two groups according to vascular access: through the femoral artery (TF-TAVI, n = 94) and through the transapical route (TA-TAVI, n = 15). Procedure endpoints, procedure success, and adverse outcomes were evaluated according to the Valve Academic Research Consortium (VARC)-2 definitions.

**Results:**

High-weight patients had a higher proportion of older (*p* < 0.001) and previous percutaneous coronary interventions (*p* = 0.026), a higher percentage of diabetes mellitus (*p* = 0.026) and frailty (*p* = 0.032), and lower glomerular filtration rate (*p* = 0.024). Procedure success was similar among the three groups. The 30-day all-cause mortality of patients with low-, middle-, and high weights was 3.7% (1/27), 5.5% (3/55), and 3.7% (1/27), respectively. In the multivariable analysis, middle- and high-weight patients exhibited similar overall mortality (middle weight vs. low weight, *p* = 0.500; high weight vs. low weight, *p* = 0.738) and similar intubation time compared with low-weight patients (9.1 ± 7.3 h vs. 8.9 ± 6.0 h vs. 8.7 ± 4.2 h in high-, middle-, and low-weight patients, respectively, *p* = 0.872). Although high-weight patients had a lower PaO_2_/FiO_2_ ratio than low-weight patients at baseline, transitional extubation, and post extubation 12th hour (*p* = 0.038, 0.030, 0.043, respectively), there were no differences for post extubation 24th hour, post extubation 48th hour, and post extubation 72nd hour (*p* = 0.856, 0.896, 0.873, respectively). Chronic lung disease [odds ratio (OR) 8.038, *p* = 0.001] rather than high weight (OR 2.768, *p* = 0.235) or middle weight (OR 2.226, *p* = 0.157) affected postoperative PaO_2_/FiO_2_ after TAVI.

**Conclusions:**

We did not find the existence of an obesity paradox after TAVI. BMI had no effect on postoperative intubation time. Patients with a higher BMI should be treated similarly without the need to deliberately extend the intubation time for TAVI.

**Supplementary Information:**

The online version contains supplementary material available at 10.1186/s13019-022-01910-x.

## Introduction

Aortic stenosis (AS) is one of the most common degenerative valvular heart diseases [1, 2]. With years of gradual development, multiple factors including genetic susceptibility, anatomical changes, and lifestyle appear to contribute to valvular development into a clinically related stage of obstruction. Due to impaired cardiac function, patients with AS have a poor prognosis and experience a high disease burden [3]. Undoubtedly, the prevalence of severe AS develops with age, affecting about 4% of the population over the age of 75 years [4, 5]. The traditional treatment has been surgical aortic valve replacement (SAVR), which involves cardiopulmonary bypass (CPB) and sternotomy. However, depending on individual comorbidities, this process may pose a high risk to some patients [6].

Transcatheter aortic valve implantation (TAVI), an interventional method by which a valve is implanted via the transfemoral artery percutaneously (TF-TAVI) or directly via the transapical route (TA-TAVI), is now a method worth considering for inoperable or high-risk surgical patients with severe AS [7, 8]. Although the TAVI technique has been greatly improved, patients undergoing TAVI are older and may have unique associated comorbidities [9].

Early extubation is associated with a good prognosis because prolonged intubation increases the risk of infections, subsequently resulting in respiratory failure [10, 11]. However, the risk factors for prolonged intubation are not fully clear. Previous research indicates that middle-weight or obese patients are more likely to be ventilated longer than low-weight patients after acute thoracic aortic dissection (ATAD) surgery [12]. Weight loss is reported to be related to a significant improvement in pulmonary function [13]. Obesity has also been considered a predictor of postoperative hypoxemia [14, 15].

Obesity is also considered an important and modifiable risk factor for cardiovascular morbidity and mortality and has been associated with greater mortality in the general population and patients with cardiovascular disease (CVD) [16, 17]. Despite their adverse effects on general health, middle-weight and high-weight patients appeared to be protected during several cardiovascular interventions [18–20]. This discrepancy was also found in patients with severe AS undergoing TAVI [18, 21–27].

This study aims to explore whether high weight also affected ventilation time and whether there is an “obesity paradox” in the prognosis of patients after TAVI, in order to obtain information that will help clinical practice.

## Subjects and methods

### Research subjects

Consecutive patients with severe symptomatic aortic stenosis (AS) who underwent TAVI between May 2018 and April 2021 at our institute and were considered inoperable or at high risk for a traditional SAVR were included [6]. The medical staff measured the height and weight of all patients on admission. To improve the sample sizes in each group, patients were classified into three groups according to 25th, 26th–75th, and 76th–99th percentiles of body mass index (BMI). Patients were considered low weight if their BMI was < 21.9 kg/m^2^, middle weight if their BMI was 21.9–27.0 kg/m^2^, and high weight if their BMI was > 27 kg/m^2^, in contrast to the previous BMI classification system used for adults in China [28]. Patients were also classified into two groups according to vascular access: through the transfemoral artery route (TF-TAVI) and through the transapical route (TA-TAVI). We determined the method that was best for patients based on current guidelines [6] and took decisions by consulting the local heart team comprising cardiac surgeons and cardiologists. Device endpoints, success rate, and related adverse events were recorded according to the Valve Academic Research Consortium (VARC)-2 definitions [29]. Patients matching one or more of the following pathological characteristics were excluded: improper aortic valve size; thrombosis of the left ventricle; active endocarditis; high risk of coronary ostia obstruction; plaque in the ascending aorta or aortic arch with active thrombus.

### Criteria

The related cardiovascular risk factors were recorded and defined as follows: hypertension as systolic blood pressure ≥ 140 mm Hg or diastolic blood pressure ≥ 90 mm Hg [30]; diabetes mellitus as fasting blood glucose ≥ 7 mmol/L or a previous diagnosis [31]; stroke based on a clinical diagnosis or CT scan [32]; hypoxemia as oxygen saturation of < 90% by pulse oximetry or available arterial oxygen < 60 mm Hg [33, 34]. Frailty was evaluated based on serum albumin levels, gait speed, grip strength, and the number of independent activities of daily living.

Informed consent was obtained from all study subjects and the anonymity of patients was preserved. The study was carried out in accordance with the ethical principles of the 1975 Declaration of Helsinki with later amendments.

### Procedural characteristics

All procedures were performed under general anesthesia and endotracheal intubation to prevent interferences in the surgical process due to poor patient compliance. TF-TAVI (VitaFlow®Transcatheter Aortic Valve, Micro Port, Shanghai, China) was performed in 94 patients by percutaneous access through the femoral artery, whereas TA-TAVI was performed via a small left anterior thoracotomy (J-Valve, JC Medical, China) in 15 patients. In both types of surgery, 100 I.E./kg body weight of heparin was used for anticoagulation, which was subsequently antagonized by protamine at a 1:1 ratio. Surgeries were performed on average by 5 well-trained surgeons. The detailed procedure is described in the Additional file 1: Video S1.

### Endpoints and follow-up

The primary outcomes were 30-day mortality and a composite of mortality to a maximum follow-up of 35 months. The secondary endpoints were perioperative pulmonary complications. All surgical survivors were monitored via telephone conversations, emails, or letters.

### Statistical analysis

The Shapiro–Wilk test was used to assess the normal distribution of the registered data. Continuous variables are presented as means ± standard deviations and were compared using one-way ANOVA (for normally distributed data) or median and 25th–75th percentile, and using Kruskal–Wallis analysis (for nonnormally distributed data). Categorical variables are presented as percentages and were compared using chi-squared or Fisher’s exact tests, as appropriate. Logistic regression analysis was conducted to determine the potential risk factors of overall mortality and hypoxemia. Factors for multivariate analysis were incorporated based on the results from univariate analysis and professional knowledge. A Kaplan–Meier curve was generated for the incidence of the primary outcome. *p* < 0.05 was considered statistically significant.

## Results

**Fig. 1:**
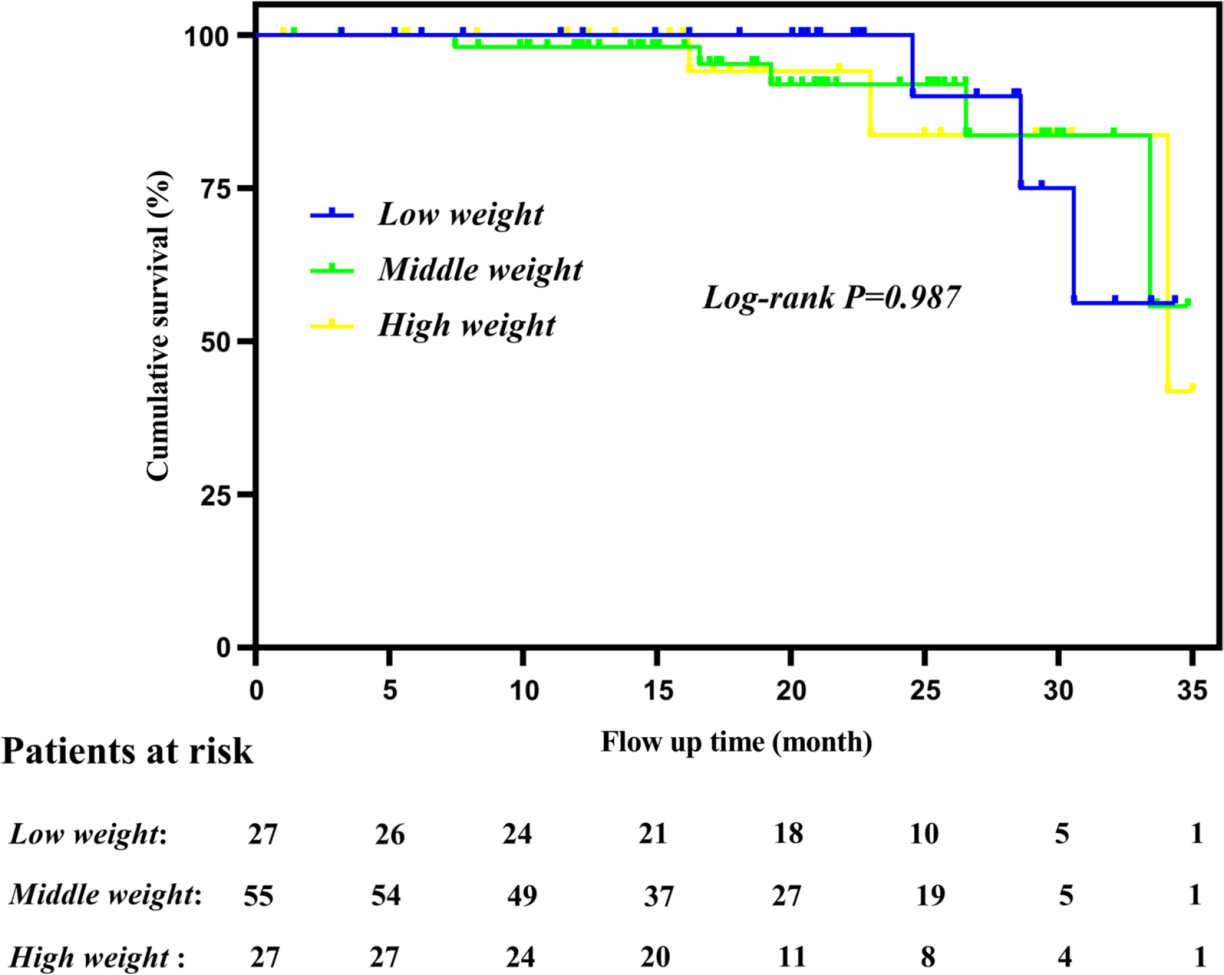
The survival curve of each BMI group

1. Baseline patient characteristics
  At our institute, 109 patients underwent TAVI; 27 patients had low weight (24.8%) with BMI of 20.3 [19.4, 21.2]; 55 patients were middle weight (50.4%) with BMI of 25.0 [23.4, 25.9]; and 27 patients were high weight (24.8%) with BMI of 30.3 [28.0, 34.4]. The transfemoral approach was used in 86.2% of cases (94 patients) and transapical approach in 13.8% of cases (15 patients). Table 1 shows the baseline clinical characteristics and pre-procedural imaging details of the study subjects. Several baseline characteristics differed between groups: high-weight patients showed a higher proportion of older subjects (*p* < 0.001) and those with previous percutaneous coronary intervention (*p* = 0.026); a higher percentage of diabetes mellitus (*p* = 0.026) and frailty (*p* = 0.032); and a lower glomerular filtration rate (*p* = 0.024). Aortic valve gradients and left ventricular ejection fraction were similar among groups.
2. Procedural details
  Procedural details are shown in Table 2. Procedure success was similar among the three BMI groups (92.6% vs. 98.2% vs. 92.6% for low-weight, middle-weight, and high-weight patients, respectively; *p* = 0.280).
3. 30-day outcomes and 35-month mortality
  The 30-day all-cause mortality was 3.7% (1/27; the patient died of concurrent liver and kidney failure) versus 5.5% (3/55; 2 patients died of acute renal failure and another died of postoperative cerebral hemorrhage due to combined nephrotic syndrome and coagulation dysfunction) versus 3.7% (1/27, the patient died of postoperative right coronary artery blockage) in low-weight, middle-weight, and high-weight patients, respectively. The 30-day complications are shown in Table 3.
  In addition to the 5 in-hospital deaths, 104 patients were discharged alive and followed up. The mean follow-up period was 18.65 ± 9.36 months (range: 0–35 months) and the 35-month mortality was 11.1% (3/27) for low-weight patients, 9.1% (5/55) for middle-weight patients, and 7.4% (2/27) for high-weight patients. Survival curves of the low-, middle-, and high-weight groups are shown in Fig. 1. The three groups of survival curves showed no significant differences between them, *p* = 0.987.
  In the univariate model, high-weight patients had decreased overall mortality compared with middle-weight patients (hazard ratio [HR] 0.887, 95% confidence interval [CI] 0.368–0.935; *p* = 0.029) (Table 4). However, in the multivariate analysis, mortality among all BMI groups was similar (HR = 1.124 and 1.231, 95% CI 0.866–1.974 and 0.920–1.995; *p* = 0.500 and 0.738 for the middle-weight and high-weight vs. the low-weight group, respectively) (Table 4). In the univariate model, BMI, age, GFR, atrial fibrillation, frailty, ejection fraction, aortic valve mean gradient, and alternative access were found to be independently associated with the 35-month all-cause mortality in this study (Table 4). In the multivariate model, diabetes mellitus, GFR, and frailty instead of higher BMI were related to overall mortality (Table 4).
4. Secondary outcomes: pulmonary complications
  Middle-weight and high-weight patients had a similar intubation time compared with low-weight patients (9.1±7.3 hours vs. 8.9±6.0 hours vs. 8.7±4.2 hours in high-, middle-, and low-weight patients, respectively, *p* = 0.872). Although high-weight patients had a lower PaO_2_/FiO_2_ ration than low-weight patients at baseline, transitional extubation, and post extubation 12th hour (*p* = 0.038, 0.030, 0.043, respectively), there were no differences at post extubation 24th hour, post extubation 48th hour, and post extubation 72th hour (*p* = 0.856, 0.896, 0.873, respectively). (Table 5).

**Table 1:** Baseline patient characteristics

Variable	Overall (n = 109)	BMI (kg/m^2^)			*p*-value
		< 21.9 (n = 27)	21.9–27.0 (n = 55)	> 27.0 (n = 27)	
Age (years)	72.0 ± 8.8	71.2 ± 7.4	72.0 ± 7.4	74.8 ± 8.8	< 0.001
Men	67 (61.5%)	18 (66.7%)	31 (56.4%)	18 (66.7%)	0.543
Body mass index (kg/m^2^)	25.0 [21.9, 27.0]	20.3 [19.4, 21.2]	25.0 [23.4, 25.9]	30.3 [28.0, 34.4]	< 0.001
New York Heart Association class III or IV	87 (79.8%)	21 (77.8%)	44 (80.0%)	22 (81.5%)	0.943
Hypertension	94 (86.2%)	21 (77.8%)	48 (87.3%)	25 (92.6%)	0.310
Diabetes mellitus	37 (33.9)	7 (25.9%)	15 (27.3%)	15 (55.6%)	0.026
Previous myocardial infarction	16 (14.7%)	5 (18.5%)	6 (10.9%)	5 (18.5%)	0.593
Previous coronary artery bypass graft	18 (16.5%)	4 (14.8%)	8 (14.5%)	6 (22.2%)	0.708
Previous percutaneous coronary intervention	28 (25.7%)	4 (14.8%)	12 (21.8%)	12 (44.4%)	0.026
Previous valve surgery	5 (4.6%)	2 (7.4%)	2 (3.6%)	1 (3.7%)	0.836
Peripheral artery disease	36 (33.0%)	8 (29.6%)	18 (32.7%)	10 (37.0%)	0.834
Previous stroke/transient ischemic attack	18 (16.5%)	4 (14.8%)	8 (14.5%)	6 (22.2%)	0.708
Chronic lung disease^a^	37 (33.9%)	4 (14.8%)	22 (40.0%)	11 (40.7%)	0.051
Glomerular filtration rate (mL/min/m^2^)	55.9 ± 25.5	61.4 ± 17.0	55.9 ± 19.6	50.0 ± 15.2	0.024
Previous pacemaker	14 (12.8%)	3 (11.1%)	9 (16.4%)	2 (7.4%)	0.570
Atrial fibrillation	27 (24.8%)	4 (14.8%)	19 (34.5%)	4 (14.8%)	0.064
Frailty^b^	31 (28.4%)	5 (18.5%)	13 (23.6%)	13 (48.1%)	0.032
Ejection fraction (%)	56.8 ± 14.9	56.0 ± 14.9	58.0 ± 13.2	56.3 ± 10.4	0.250
Aortic valve area (cm^2^)	0.63 ± 0.16	0.63 ± 0.15	0.63 ± 0.16	0.64 ± 0.15	0.998
Aortic valve mean gradient (mm Hg)	45.4 ± 13.6	46.0 ± 13.3	44.9 ± 14.2	45.4 ± 13.0	0.386
Aortic valve maximal gradient (mm Hg)	76.2 ± 21.4	77.1 ± 20.8	74.2 ± 22.2	77.0 ± 20.5	0.286
CT mean annulus diameter (mm)	24.3 ± 2.7	24.3 ± 2.8	24.4 ± 2.5	24.2 ± 2.5	0.980

Data are expressed as mean ± standard deviations (SD), median ( first quartile, third quartile) or number (%). Chi-square or Fisher test for categorical variables and t text or wilcoxon test for continuous variables

^a^Interstitial lung disease or chronic obstructive pulmonary disease or asthma

^b^Assessed based on serum albumin, gait speed, grip strength, and number of independent activities of daily leavingSuggested criteria for the diagnosis of frailty included 5-min walking time, grip strength, BMI < 20 kg/m^2^ and/or weight loss of 5 kg/year, serum albumin < 3.5 g/dL, and cognitive impairment or dementia

**Table 2:** Procedural details

Variable	BMI (kg/m^2^)			*p*-value
	< 21.9 (n = 27)	21.9–27.0 (n = 55)	> 27.0 (n = 27)	
Implanted valve				1.000
VitaFlow®^a^	23 (85.2%)	48 (87.3%)	23 (85.2%)	
J-Valve^b^	4 (14.8%)	7 (12.7%)	4 (14.8%)	
Implanted valve size (mm)				
21 (VitaFlow®)	8 (29.6%)	16 (29.1%)	6 (22.2%)	0.532
24 (VitaFlow®)	14 (51.9%)	25 (45.4%)	12 (44.5%)	
27 (VitaFlow®)	5 (18.5%)	14 (25.5%)	7 (25.9%)	
30 (VitaFlow®)	0 (0.0%)	0 (0.0%)	2 (7.4%)	
Vascular access				
Transfemoral	22 (81.5%)	48 (87.3%)	24 (88.9%)	0.762
Transapical	5 (18.5%)	7 (12.7%)	3 (11.1%)	
Device success	25 (92.6%)	54 (98.2%)	25 (92.6%)	0.280
2nd valve	2 (7.4%)	2 (3.6%)	1 (3.7%)	0.836
Postdilatation	3 (11.1%)	6 (10.9%)	4 (14.8%)	0.936
Valve embolization	1 (3.7%)	0 (0.0%)	0 (0.0%)	0.495
Fluoroscopy time (min)	17.8 ± 7.1	15.9 ± 7.0	16.2 ± 8.8	0.638
Total contrast used (ml)	85.9 ± 47.6	89.2 ± 42.8	90.1 ± 40.2	0.236
TEE postprocedural PVL				
None/trace	22 (81.5%)	43 (78.2%)	21 (77.8%)	1.000
Mild	5 (18.5%)	11 (20.0%)	5 (18.5%)	
Moderate	0 (0.0%)	1 (1.8%)	1 (3.7%)	
Severe	0 (0.0%)	0 (0.0%)	0 (0.0%)	
Postprocedural aortic valve gradient (mm Hg)	8.5 ± 4.6	9.5 ± 5.0	8.5 ± 5.0	0.383

Data are expressed as mean ± standard deviations (SD) or number (%). Chi-square or Fisher test for categorical variables and t text for continuous variables

*PVL* perivalvular leak, *TEE* transesophageal echocardiography

^a^VitaFlow®Transcatheter Aortic Valve, Micro Port, ShangHai, China

^b^J-Valve Transcatheter Aortic Valve, JC Medical, China

**Table 3:** Clinical 30-day outcome

Variable	Overall (n = 109)	BMI (kg/m^2^)		
		< 21.9 (n = 27)	21.9–27.0 (n = 55)	> 27.0 (n = 27)
30-day Mortality	5 (4.6%)	1 (3.7%)	3 (5.5%)	1 (3.7%)
Cerebrovascular accident/transient ischemic attack	3 (2.8%)	1 (3.7%)	1 (1.8%)	1 (3.7%)
Myocardial infarction	2 (1.8%)	0 (0.0%)	1 (1.8%)	1 (3.7%)
Respiratory failure	7 (6.4%)	2 (7.4%)	3 (5.5%)	2 (7.4%)
Cardiogenic shock	3 (2.8%)	1 (3.7%)	2 (3.6%)	0 (0.0%)
Cardiac tamponade	1 (0.9%)	1 (3.7%)	0 (0.0%)	0 (0.0%)
Major bleeding	2 (1.8%)	1 (3.7%)	1 (1.8%)	0 (0.0%)
Major vascular complications	4 (3.7%)	1 (3.7%)	2 (3.6%)	1 (3.7%)
Minor vascular complications	8 (7.3%)	4 (14.8%)	3 (5.5%)	1 (3.7%)
New permanent pacemaker implantation	7 (6.4%)	2 (7.4%)	3 (5.5%)	2 (7.4%)
Acute kidney injury stage 3	13 (11.9%)	4 (14.8%)	7 (12.7%)	2 (7.4%)
New York Heart Association functional class	1.83 ± 0.7	1.81 ± 0.9	1.83 ± 0.9	1.80 ± 0.7

Data are expressed as mean ± standard deviations (SD) or number (%)

Chi-square or Fisher test for categorical variables and t text for continuous variables

**Table 4:** Univariate and multivariate cox proportional hazard analysis of overall mortality

Variable	Univariate analysis			Multivariate analysis		
	HR	95%CI	*p*	HR	95%CI	*p*
BMI (categorical)						
Middle versus Low	1.662	0.365–6.985	0.438	1.124	0.866–1.974	0.500
High versus Low	0.887	0.368–0.935	0.029	1.231	0.920–1.995	0.738
BMI^a^ (kg/m^2^)	0.959	0.922–0.968	0.032			
Age (years)	3.119	1.192–5.106	0.043	1.168	0.993–1.867	0.323
Male	1.032	0.988–1.996	0.788	0.855	0.733–1.457	0.534
Diabetes mellitus	0.932	0.329–2.503	0.779	3.930	1.995–4.885	0.034
Chronic lung disease	1.986	0.392–2.831	0.409	1.306	0.156–1.887	0.460
CAD	1.988	0.959–2.887	0.569	1.004	0.566–1.661	0.660
Previous MI	0.925	0.780–2.944	0.099			
Previous CABG or valve surgery	0.317	0.102–0.980	0.328			
Previous stroke/TIA	0.878	0.884–1.488	0.587			
GFR (mL/min/m^2^)	0.898	0.877–1.219	< 0.001	3.006	1.876–3.301	0.046
Atrial fibrillation	1.318	1.259–2.916	0.04	1.558	0.885–2.432	0.120
Frailty	3.125	2.035–3.152	< 0.001	3.825	1.968–4.730	0.003
Ejection fraction	0.958	0.881–0.976	0.01	1.678	0.807–1.806	0.063
Aortic valve mean gradient (mm Hg)	1.798	1.195–1.989	0.004	1.806	1.606–1.965	0.122
Aortic valve area (cm^2^)	1.006	0.625–1.986	0.763			
Valve type: VitaFlow®/J-Valve	1.160	0.914–1.246	0.108	1.803	0.875–1.954	0.098
Alternative access: Transfemoral/Transapical	2.940	1.338–2.957	< 0.001	1.551	1.022–1.997	0.088

Covariates included in the multivariate analysis: BMI categories, age, gender, diabetes mellitus, chronic lung disease, coronary artery disease, glomerular filtration rate, atrial fibrillation, frailty, ejection fraction, aortic valve mean gradient, valve type, and alternative access

*HR* hazard ratio, *CI* confidence interval, *BMI* body mass index, *CAD* coronary artery disease, *MI* myocardial infarction, *CABG* coronary artery bypass graft, *TIA* transient ischemic attack, *GFR* glomerular filtration rate

^a^BMI as linear variable; hazard ratio per 1 kg/m^2^ increment

**Table 5:** Pulmonary complications

Variable	Overall (n = 109)	BMI (kg/m^2^)			*p*-value
		< 21.9 (n = 27)	21.9–27.0 (n = 55)	> 27.0 (n = 27)	
Intubation time (hours)	9.1 ± 6.9	8.7 ± 4.2	8.9 ± 6.0	9.1 ± 7.3	0.872
Baseline PaO_2_/FiO_2_ (mmHg)	446.67 ± 50.95	451.90 ± 58.35	437.72 ± 78.85	379.88 ± 73.35	0.038
Transitional extubation, PaO_2_/FiO_2_ (mmHg)	386.67 ± 67.88	426.84 ± 59.54	366.85 ± 69.78	300.65 ± 70.40	0.030
Postextubation 12th hour, PaO_2_/FiO_2_ (mmHg)	417.58 ± 55.15	438.80 ± 65.98	410.10 ± 45.58	345.20 ± 50.35	0.043
Postextubation 24th hour, PaO_2_/FiO_2_ (mmHg)	418.18 ± 64.24	425.02 ± 29.54	405.56 ± 58.85	420.80 ± 69.52	0.856
Postextubation 48th hour, PaO_2_/FiO_2_ (mmHg)	426.06 ± 36.06	442.89 ± 60.38	384.05 ± 36.85	423.65 ± 49.97	0.896
Postextubation 72th hour, PaO_2_/FiO_2_ (mmHg)	393.67 ± 46.06	407.28 ± 45.58	405.38 ± 35.54	392.59 ± 41.88	0.873

Data are expressed as mean ± standard deviations (SD)

T text for continuous variables

Chronic lung disease (OR 8.038, *p* = 0.001) rather than high weight (OR 2.768, *p* = 0.235) or middle weight (OR 2.226, *p* = 0.157) affected the postoperative PaO_2_/FiO_2_ ratio after TAVI (Table 6).

**Table 6:** Logistic regression of hypoxemia

Variable	Odds ratio	95% Confidence interval	Overall *p*-value
BMI (kg/m^2^)			
BMI: Low	1.000		
BMI: Middle	2.226	0.996–11.875	0.157
BMI: High	2.768	0.998–13.085	0.235
Chronic lung disease			
No	1.000		
Yes	8.038	3.682–38.096	0.001^a^

*BMI* body mass index

^a^Statistically significant

## Discussion

In this study, the baseline characteristics of obese patients undergoing TAVI varied; there was a higher proportion of females and higher prevalence of diabetes mellitus, coronary artery disease, chronic lung disease and frailty, and lower estimated GFR. However, the rates of procedural complications and device success were similar among all BMI groups.

### Obesity paradox in the univariate model

In the general population, as the BMI increases above 30 kg/m^2^, the risk of developing CVD increases and the risk of mortality is higher [35]. Nonetheless, it is often observed that obese individuals with chronic diseases have a reduced risk of mortality compared with nonobese individuals with similar disease characteristics. The positive effect of increased BMI following these interventions has been called the “obesity paradox.” This paradox has been described in several disease categories, including CVD, diabetes mellitus, and chronic kidney disease [36–38]. The “obesity paradox” has been reported mainly in patients with heart failure, acute coronary syndrome, and following percutaneous and surgical coronary interventions [18–20, 36, 38]. Some studies have explained that this protective effect is because the soluble tumor necrosis factor (TNF)-α receptors in adipose tissues neutralize the adverse effects of TNF-α and decreases the mortality of patients with chronic inflammatory diseases such as CVD [39]. In addition, higher lipoproteins in circulation may also bind and reduce the role of lipopolysaccharides in stimulating the release of inflammatory cytokines [38].

There is also evidence for the obesity paradox in high-risk patient groups after TAVI [21, 26]. Konigstein et al. reported that after adjusting baseline characteristics, increased BMI was independently related to the improvement in survival after TAVI [21]. Conclusions of the data analysis of the large FRANCE 2 Registry that included 3072 patients were also consistent with the obesity paradox after TAVI [26]. In our univariate model, high weight was found to be closely related to increased survival rates, which was in line with the obesity paradox.

### Disappearance of the obesity paradox in the multivariate model

In the multivariate model, the overall mortality rate was found to be similar among the three BMI groups after adjusting for different baseline characteristics including diabetes mellitus, GFR, and frailty. Our findings were contradictory to those of previous studies; according to our multivariable model, there was no obesity paradox after TAVI and the overall midterm survival rates were similar among all BMI groups (Fig 1). Previous studies have explained this discrepancy because the baseline frailty factor was not included in their research and analysis models. The updated VARC-2 document defines frailty as slowness, weakness, exhaustion, wasting and malnutrition, inactivity, and loss of independence, and emphasizes that frailty is the most important feature in current risk models [29] and is a strong predictor of mortality and adverse effects after cardiac surgery and TAVI [40–42]. Indeed, in this study, frailty was a strong predictor of overall mortality in both the univariate and multivariate models (HR 3.825; *p* = 0.003; Table 4). Meanwhile, 48.1% of high-weight patients were frail versus 18.5% of low-weight patients (*p* = 0.032), which might be a possible confounder in resisting the occurrence of the obesity paradox. In addition, high-weight patients in the current study were significantly older, had lower GFR and higher incidences of previous percutaneous coronary intervention, and prevalence of diabetes mellitus, all of which are possible confounding factors that might provide an explanation for the loss of the protective effect of high weight on mortality.

### Effect of BMI on pulmonary complications

Many studies have consistently reported that patients who are obese are more likely to develop severe hypoxemia after surgery due to the significant decline in lung compliance. Due to their abnormal BMI, certain clinical features of patients who are obese may require special management during the perioperative period, such as higher doses of medications, greater tidal volume, and higher airway pressure to ensure adequate postoperative ventilation. Therefore, there is a significant increase in dyspnea in individuals who are obese people. Additionally, respiratory resistance increases in high-weight individuals, and the mechanism of acute respiratory distress syndrome may be attributed to an imbalance in anti-inflammatory and pro-inflammatory cytokine levels and in the oxidant and antioxidant levels [43–46]. Most patients who are obese suffer from chronic and excessive inflammation and oxidative stress [43, 44]. When pro-inflammatory signaling pathways are significantly upregulated, patients who are obese are prone to produce more abnormal cytokine products and acute-phase reactants. Furthermore, the production of pro-inflammatory cytokines and mediators is known to increase with weight gain [45, 46]. Moreover, high weight can increase oxidative stress and lead to an increase in reactive oxygen products, which may lead to direct damage of cellular membranes, cause monocyte cellular adhesion, and the release of chemotactic factors and vasoactive substances. The above processes are obvious especially while undergoing CPB. However, in this study, although patients with high BMI had lower PaO_2_/FiO_2_ than low-weight patients at pre- and early postoperative time (baseline, transitional extubation, and post extubation 12th hour), high weight did not have a significant effect on severe postoperative hypoxemia and longer intubation time in our multiple regression model. An explanation for this divergence may be that patients with TAVI without CPB did not have a marked activation of the signaling pathways discussed above.

## Limitations

There may have been selection bias owing to the retrospective, single-center nature of this study. Moreover, the small sample of this study might have limited our findings and conclusions. Secondly, operator experience can be an important determinant of results. Furthermore, differences in a longer-term prognosis of patients are needed. Lastly, a more comprehensive evaluation of the prognostic value of BMI as a predictor of clinical results after TAVI is required.

## Conclusions

We found no “obesity paradox” after TAVI, which is a finding that is in contrast with previous reports that claim a protective effect from high BMI after TAVI, likely because patients with high BMI were at a higher risk of being older; of having lower GFR and a higher prevalence of previous percutaneous coronary intervention, diabetes mellitus, and frailty. Instead, patients who were obese had a lower oxygenation index (PaO_2_/FiO_2)_ in the early postoperative period without a longer intubation time, which reminds us of the patient population of high-BMI patients after TAVI.

## Supplementary Information

**Additional file 1:** Video of TAVI procedure.

## Data Availability

Data sharing not applicable to this article as no data sets were generated or analyzed during the current study.

## Abbreviations

AS: Aortic stenosis
TAVI: Transcatheter aortic valve implantation
BMI: Body mass index
PVL: Perivalvular leak
TEE: Transesophageal echocardiography
GFR: Glomerular filtration rate
